# DNA methylation epi-signature is associated with two molecularly and phenotypically distinct clinical subtypes of Phelan-McDermid syndrome

**DOI:** 10.1186/s13148-020-00990-7

**Published:** 2021-01-06

**Authors:** L. C. Schenkel, E. Aref-Eshghi, K. Rooney, J. Kerkhof, M. A. Levy, H. McConkey, R. C. Rogers, K. Phelan, S. M. Sarasua, L. Jain, R. Pauly, L. Boccuto, B. DuPont, G. Cappuccio, N. Brunetti-Pierri, C. E. Schwartz, B. Sadikovic

**Affiliations:** 1 Molecular Genetics Laboratory, Molecular Diagnostics Division, London Health Sciences Centre, London, ON N6A5W9 Canada; 2 Department of Pathology and Laboratory Medicine, Western University, London, ON N6A3K7 Canada; 3 Greenwood Genetic Center, Greenwood, SC 29646 USA; 4 Greenville Office, Greenwood Genetic Center, Greenville, SC 29605 USA; 5 Genetics Laboratory, Florida Cancer Specialists and Research Institute, Fort Myers, FL 33816 USA; 6 Clemson University, Clemson, SC 29634 USA; 7 Department of Translational Medicine, University Federico II, 80131 Naples, NA Italy; 8 Telethon Institute of Genetics and Medicine, Pozzuoli, NA Italy

**Keywords:** DNA methylation, Microdeletion, Epi-signature, BRD1, Phelan-McDermid syndrome

## Abstract

**Background:**

Phelan-McDermid syndrome is characterized by a range of neurodevelopmental phenotypes with incomplete penetrance and variable expressivity. It is caused by a variable size and breakpoint microdeletions in the distal long arm of chromosome 22, referred to as 22q13.3 deletion syndrome, including the *SHANK3* gene. Genetic defects in a growing number of neurodevelopmental genes have been shown to cause genome-wide disruptions in epigenomic profiles referred to as epi-signatures in affected individuals.

**Results:**

In this study we assessed genome-wide DNA methylation profiles in a cohort of 22 individuals with Phelan-McDermid syndrome, including 11 individuals with large (2 to 5.8 Mb) 22q13.3 deletions, 10 with small deletions (< 1 Mb) or intragenic variants in *SHANK3* and one mosaic case. We describe a novel genome-wide DNA methylation epi-signature in a subset of individuals with Phelan-McDermid syndrome.

**Conclusion:**

We identified the critical region including the *BRD1* gene as responsible for the Phelan-McDermid syndrome epi-signature. Metabolomic profiles of individuals with the DNA methylation epi-signature showed significantly different metabolomic profiles indicating evidence of two molecularly and phenotypically distinct clinical subtypes of Phelan-McDermid syndrome.

## Background

Phelan-McDermid syndrome (PHMDS) is characterized by developmental delay, absent or impaired speech, neonatal hypotonia, autistic behaviors and mild dysmorphic features [1, 2]. It is caused by a contiguous gene deletion of the distal long arm of chromosome 22 (also referred to as 22q13.3 deletion syndrome). This loss of genetic material can be caused by a terminal or interstitial deletion of chromosome 22, an unbalanced translocation that can be inherited or de novo, or from other complex structural rearrangements involving chromosome 22 [2]. The size of the 22q13.3 deletion in PHMDS ranges from < 50 kb to > 9 Mb, including complete or partial deletion of the *SHANK3* (SH3 and multiple ankyrin repeat domains 3) gene in virtually all cases.

A limited number of individuals with loss-of-function intragenic variants in the *SHANK3* gene have been reported. Most individuals with *SHANK3* intragenic variants have been described as having autism spectrum disorder (ASD) and/or intellectual disability [3–6]. A recent study had phenotypically characterized 17 individuals with pathogenic variants in *SHANK3*, demonstrating that *SHANK3* intragenic pathogenic variants are sufficient to cause a broad range of features associated with PHMDS [7]. However, even though intellectual disability and ASD were present in the majority of individuals with *SHANK3* mutations, speech impairment and motor deficits were less severe than in 22q13 deletions, and renal abnormalities were absent [7]. Taken together, there is compelling evidence, suggesting that *SHANK3* haploinsufficiency is responsible for the majority of neurological features, i.e., intellectual disability and autism, but not the full spectrum of phenotypes observed in PHMDS [8–10]. There are at least two cases reported with 22q13 interstitial deletions sparing *SHANK3* and phenotypes similar to those in PHMDS [11], suggesting an independent role for genes in these genomic regions. Another candidate gene located near *SHANK3*, and deleted in most cases of the syndrome, is the autism-linked gene *IB2* (islet brain 2) that plays an important role in synaptic transmission and neuronal morphology [12].

Genotype–phenotype relationships in PHMDS have been extensively studied [13–17]. For most phenotypes, the individuals with the smallest terminal deletions (22q13.33) were less severely affected than those with the largest terminal deletions (22q13.2), except for ASD which is commonly associated with smaller deletions. These studies suggested that the effect of *SHANK3*, a gene associated with ASD, may be attenuated as the deletion size increases and additional genes are deleted. This may reflect the difficulty in diagnosing ASD in individuals that are more severely affected. In addition, these studies mapped a large number of potential candidate genes within 22q13 region, including genes associated with dysmorphic features, speech and behavior. Taken together, these studies emphasize that PHMDS is a clinically heterogenous syndrome caused by haploinsufficiency of multiple candidate genes. The large range of deletion sizes observed resulting in a large range of phenotypical features, together with the lack of knowledge of all candidate genes within the deleted region, complicates the clinical and molecular diagnosis and/or prognosis for these individuals.

DNA methylation is one of the most widely studied epigenetic mechanisms and is involved in many physiological and disease mechanisms through control of gene expression and genomic stability. The establishment of the DNA methylation pattern occurs early during embryonic development and varies significantly across different tissues. Genomic DNA methylation profiles in peripheral blood, referred to as DNA methylation epi-signatures, have been associated with many human traits, including age, sex and disease status [18–21]. More recently, we and others have reported evidence of highly sensitive and specific peripheral blood epi-signatures associated with genetic mutations in a growing number of genes and genetic syndromes associated with developmental delay/intellectual disabilities and multiple congenital anomalies [22–25]. In addition, microarray-based genomic DNA methylation assessment has enabled simultaneous detection of imprinted disorders and Fragile X syndrome, which is often within the spectrum of differential diagnoses of these neurodevelopmental syndromes [26, 27].

In this study we assessed genome-wide DNA methylation profiles in a cohort of individuals with PHMDS, including individuals with large (2 to 5.8 Mb) 22q13.3 deletions, with small deletions (< 1 Mb) or with intragenic variants in *SHANK3*. Given the molecular and phenotypic complexity of this disease, we hypothesized a differentiating DNA methylation epi-signature that would enable assessment of variants of unknown clinical significance, such as smaller deletions at 22q13 or *SHANK3* variants, as well as diagnosis of ambiguous clinical cases. We describe a highly sensitive and specific DNA methylation signature associated with large 22q13.3 deletions. This signature is not evident in individuals with small deletions or *SHANK3* intragenic variants, hence differentiating these as separate molecular entities. Using these data, we build a classification algorithm for PHMDS and demonstrate its ability to specifically identify individuals with PHMDS-large deletions differentiating them from other neurodevelopmental conditions with overlapping phenotypes, as well as other genetic conditions with recognized DNA methylation epi-signature. Metabolic profiling of cells from individuals with large 22q13 deletions revealed a functional impact of the disrupted DNA methylation by showing, among other features, reduced capacity of cells to produce energy utilizing high-efficiency aerobic pathways, decreased ability to adjust to metabolic stress and abnormal response to hormones and cytokines regulating growth and inflammation, as compared to controls or cells with small 22q13 deletions or *SHANK3* variants. The metabolic findings provide in vitro validation of the hypothesis that abnormal genome-wide DNA methylation and large 22q13 deletions in PHMDS lead to a larger number of disrupted pathways and more severe clinical phenotypes than in cases with small deletions or *SHANK3* variants and suggest the existence of distinguishable subtypes of PHMDS.

## Results

### Genetic profiling of individuals with Phelan-McDermid syndrome

This study included 22 subjects with confirmed clinical features consistent with PHMDS and molecular/cytogenetic diagnoses of PHMDS.r These subjects had previous chromosome microarray testing performed for 22q13 genomic deletion detection, as well as Sanger sequencing of *SHANK3* gene in individuals without 22q13 deletion. The molecular description at diagnosis and demographics of all subjects is shown in Table 1. Eleven individuals carried a large deletion at chr22q13 (referred to as PHMDS-Large Del), spanning a region > 1 Mb and including *SHANK3* gene region. Of the remaining individuals (included in the PHMDS-Small Del/Mut cohort), five presented with smaller deletions, ranging from 0.06 to 0.92 Mb, including *SHANK3* region; five individuals had *SHANK3* intragenic mutations; and one individual was mosaic for a large deletion at chr22q13.

**Table 1:** Demographic and molecular characteristics of Phelan-McDermid cohort

Molecular cohort	ID	Age	Sex	Molecular description #
PHMDS Small Del/Mut	MS2449	14	M	NM_001372044.1(SHANK3):c.1339dup, p.(Arg447Profs*41)
PHMDS Small Del/Mut	MS2452	15	F	NM_001372044.1(SHANK3):c.4113_4114del, p.(Glu1371Aspfs*15)
PHMDS Small Del/Mut	MS2453	15	F	NM_001372044.1(SHANK3):c.4113_4114del, p.(Glu1371Aspfs*15)
PHMDS-Small Del/Mut	MS2455	19	F	NM_001372044.1(SHANK3):c.3472_3473del, p.(Gly1158Profs*212)
PHMDS-Small Del/Mut	MS2457	4	M	NM_001372044.1(SHANK3_v001):c.4086_4087del, p.(Ala1363Profs*7)
PHMDS-Small Del/Mut	MS2676	10	M	Deletion size 0.013 Mb (SHANK3 deletion)
PHMDS-Small Del/Mut	MS2675	10	M	Deletion size 0.013 Mb (SHANK3 deletion)
PHMDS Small Del/Mut	MS2450	10	F	Deletion size 0.06 Mb
PHMDS-Small Del/Mut	MS2456	18	M	Deletion size 0.68 Mb
PHMDS Small Del/Mut	MS2454	6	F	Deletion size 0.92 Mb
PHMDS-Large Del**	MS2463	3	M	Deletion size 1.95 Mb
PHMDS-Large Del**	MS2444	15	M	Deletion size 2.32 Mb
PHMDS-Large Del**	MS2443	17	F	Deletion size 2.54 Mb
PHMDS-Large Del**	MS2441	17	M	Deletion size 3.02 Mb
PHMDS-Large Del**	MS2445	3	F	Deletion size 3.46 Mb
PHMDS-Large Del**	MS2447	3	F	Deletion size 4.36 Mb
PHMDS-Large Del**	MS2442	3	F	Deletion size 4.74 Mb
PHMDS-Large Del**	MS2440	10	F	Deletion size 4.78 Mb
PHMDS-Large Del**	MS2458	8	F	Deletion size 5.16 Mb
PHMDS-Large Del**	MS2465	5	M	Deletion size 5.60 Mb
PHMDS-Large Del**	MS2462	9	M	Deletion size 5.90 Mb
PHMDS-Small Del/Mut *	MS2460	13	M	22q13 [9]/normal [21] Mosaic

PHMDS refers to individuals with > 1 Mb 22q13 deletion. PHMDS Small Del/Mut refers to individuals with small (< 1 Mb) deletions and *SHANK3* mutations. The genomic build for variant descriptions is Hg19. ^#^Molecular description was obtained from initial molecular/cytogenetics testing performed in this cohort at diagnosis. Detailed genomic locations of deletions are shown in Table 2. *Mosaic case with typical deletion included in the PHMDS Small Del/Mut cohort as it was not detected by epi-signature. **Samples with epi-signature

### Clinical features

Clinical data were collected from medical records and from the PHMDS International Registry and are listed in Table 2. The most recurrent features showed no major differences between the two cohorts of individuals and included ASD or autistic traits (reported in almost all individuals) and other behavioral or mental disorders. Other common neurological traits, such as seizures, sleep problems and hypotonia, were also fairly equally distributed between the two cohorts, while symptoms affecting neurosensory regulation or different systems (gastrointestinal, kidney, muscular/skeletal) appear to be more represented in the cohort with large 22q13 deletions, although for some of these features clinical information was available only for a limited number of individuals.

**Table 2:** Clinical features in individuals with PHMDS

	PHMDS large Del (*n* = 11)	PHMDS Small Del/Mut (*n* = 11)
Seizures	6 (54.5%)	7 (63.6%)
Sleep problems	7 (63.6%)	8 (72.7%)
Hypotonia	6 (54.5%)	4 (36.4%)
ASD or autistic traits	11 (100%)	9 (81.8%)
Other behavioral/mental disorders	6 (54.5%)	7 (63.6%)
Gastrointestinal or kidney issues	5 (45.4%)	1 (9.1%)
Abnormal thermoregulation	4 (36.4%)	1 (9.1%)
Higher tolerance to pain	5 (45.4%)	1 (9.1%)
Muscular/skeletal conditions	4 (36.4%)	0
Ventricular septal defect	0	2 (18.2%)

### DNA methylation epi-signature in PHMDS

**Fig. 1:**
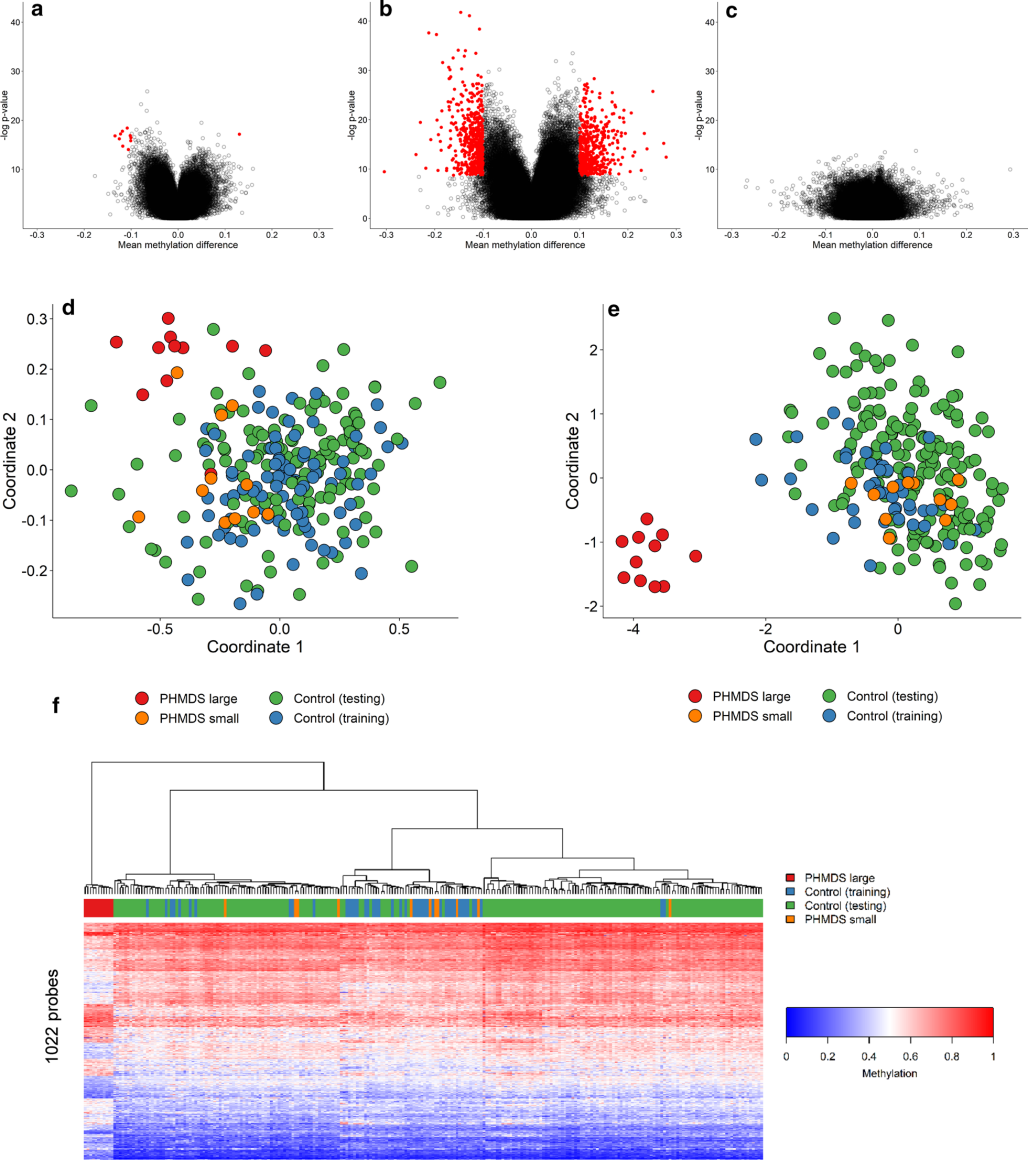
PHMDS epi-signature. Microarray probes with differing methylation in PHMDS samples were identified by comparing to control samples either all PHMDS samples (**a**), PHMDS samples with large deletions (**b**) or PHMDS samples with small deletions (**c**). Each dot represents one probe and significantly changed probes are in red. No significantly changed were found for the PHDMS small deletion samples. **d** The 11 probes identified using all PHMDS samples were used for MDS analysis and were not able to clearly distinguish between PHMDS samples and controls. Besides the controls used for probe selection (training), additional controls from our database (testing) were tested. **e** The 1022 probes identified using the PHMDS large deletion samples were able to clearly distinguish between PHMDS and control samples by MDS. **f** Hierarchical clustering using the same probes and samples as in **e**. A distinct cluster mainly representing hypermethylation events, and the other 11 subjects cluster with controls. The top lane in the heatmap indicates the phenotype. The heatmap color scale from blue to red represents the range of the methylation levels (beta values) between 0 and 1

Genome-wide DNA methylation analysis was performed on peripheral blood DNA from the 22 subjects with confirmed clinical and molecular diagnoses of PHMDS using Illumina Infinium EPIC arrays. The PHMDS samples all had fewer than 1000 failed probes, passing the quality control requirements. A comparison was made between PHMDS cohorts and age-, sex- and batch-matched controls in a ratio of 4:1 (4 controls for each PHMDS samples). Figure 1a–c shows initial comparison using all PHMDS samples, using PHMDS samples with large deletions (> 1 Mb), and using PHMDS samples with small deletions (< 1 Mb). Results of this analysis showed that only the PHMDS-Large Del cohort showed significant methylation difference when compared to controls. Whereas analysis including all PHMDS individuals showed only 11 probes with significant methylation differences (Fig. 1a), analysis of the PHMDS-Large Del cohort analysis showed 1022 differentially methylated probes (Fig. 1b). PHMDS-Small Del cohort not shows any methylation differences from control samples (Fig. 1c). Thus, identification of the epi-signature and creation of a classification model were performed for the PHMDS-Large Del cohort only (*n* = 11). Briefly, analysis including PHMDS-Large Del cases and matched controls identified 1022 probes with a minimum of 10% methylation difference between the two cohorts and a multiple testing corrected *p* value < 0.01 (limma multivariable regression modeling), adjusted for blood cell type compositions (Additional file 1: Table S1). Information about controls, including sex, age and blood cell compositions, is shown in Additional file 2: Table S2. Hierarchical clustering and multiple dimensional scaling (MDS) demonstrate that the 1022 selected probes strongly separate the PHMDS-Large Del individuals from a larger cohort of controls (Fig. 1e, f). PHMDS cases with small deletions or intragenic mutations in SHANK3 gene do not present with the epi-signature, clustering together with the control group (Fig. 1e, f). As for comparison, the 11 probes identified using all PHMDS samples were used for MDS analysis and were not able to clearly distinguish between PHMDS samples and controls (Fig. 1d).

Cross-validation using PHMDS-Large Del samples was performed to validate sensitivity of our epi-signature. For each round of validation, nine of the eleven PHMDS large deletion samples were used for probe selection along with matched controls and the remaining two PHMDS large deletion samples were saved for testing. Multidimensional scaling showed that every time the two testing samples clustered with the other PHMDS sample (Additional file 3: Figure S1).

### Identification of differentially methylated regions (DMR)

**Fig. 2:**
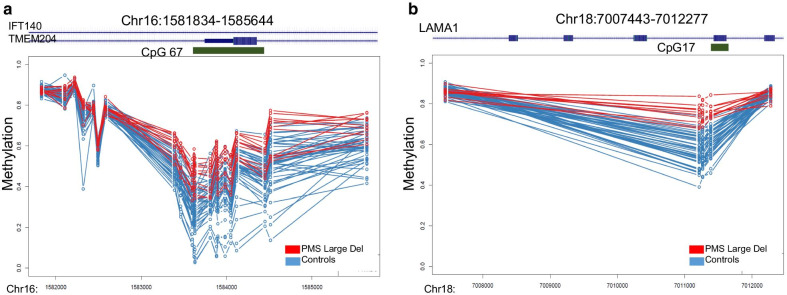
Differentially methylated regions in PHMDS-Large Del epi-signature. **a** Methylation levels across 15 differentially methylated probes in the IFT140 gene (intronic CPG island) show hypermethylation in PHMDS patients. **b** Methylation levels across 4 differentially methylated probes in the LAMA1 gene show hypermethylation in PHMDS patients. X-axis, genomic coordinate; Y-axis, DNA methylation levels between 0 and 1; circles, DNA methylation level for every individual at one CpG site; red lines, PHMDS-Large Del patients, blue lines, controls. Methylation patterns in all DMRs are provided in Additional file 4: Table S3.

Using the DMRcate algorithm [28], we prioritized a total of 29 DMRs for the PHMDS-Large Del epi-signature based on the following criteria: three or more probes less than 1 kb apart, > 10% average regional methylation change and a false discovery rate (FDR) of < 0.01, adjusted for blood cell-type compositions (Additional file 4: Table S3, Fig. 2). The vast majority of the DMRs identified in the PHMDS-Large Del epi-signature involved hypermethylation events (*n* = 24). These regions are located across multiple chromosomes and could involve clinically relevant genes. Notably, some of these genes are involved in neurotransmission regulation (*ARPP21*, OMIM# 605488), brain development (*EBF4*, OMIM #609935), and few of them are associated with other Mendelian conditions (*IFT140,* OMIM# 614620 and *LAMA1* OMIM#150320). Gene ontology enrichment analysis was performed for the DMR (Additional file 3: Figure S2). This analysis showed enrichment for pathways involved in cell morphogenesis, neural tube development, forebrain development and intracellular signal transduction. However, the number of DMRs is small to result in a statistically significant enrichment.

### Development of the MVP score for Phelan-McDermid syndrome

**Fig. 3:**
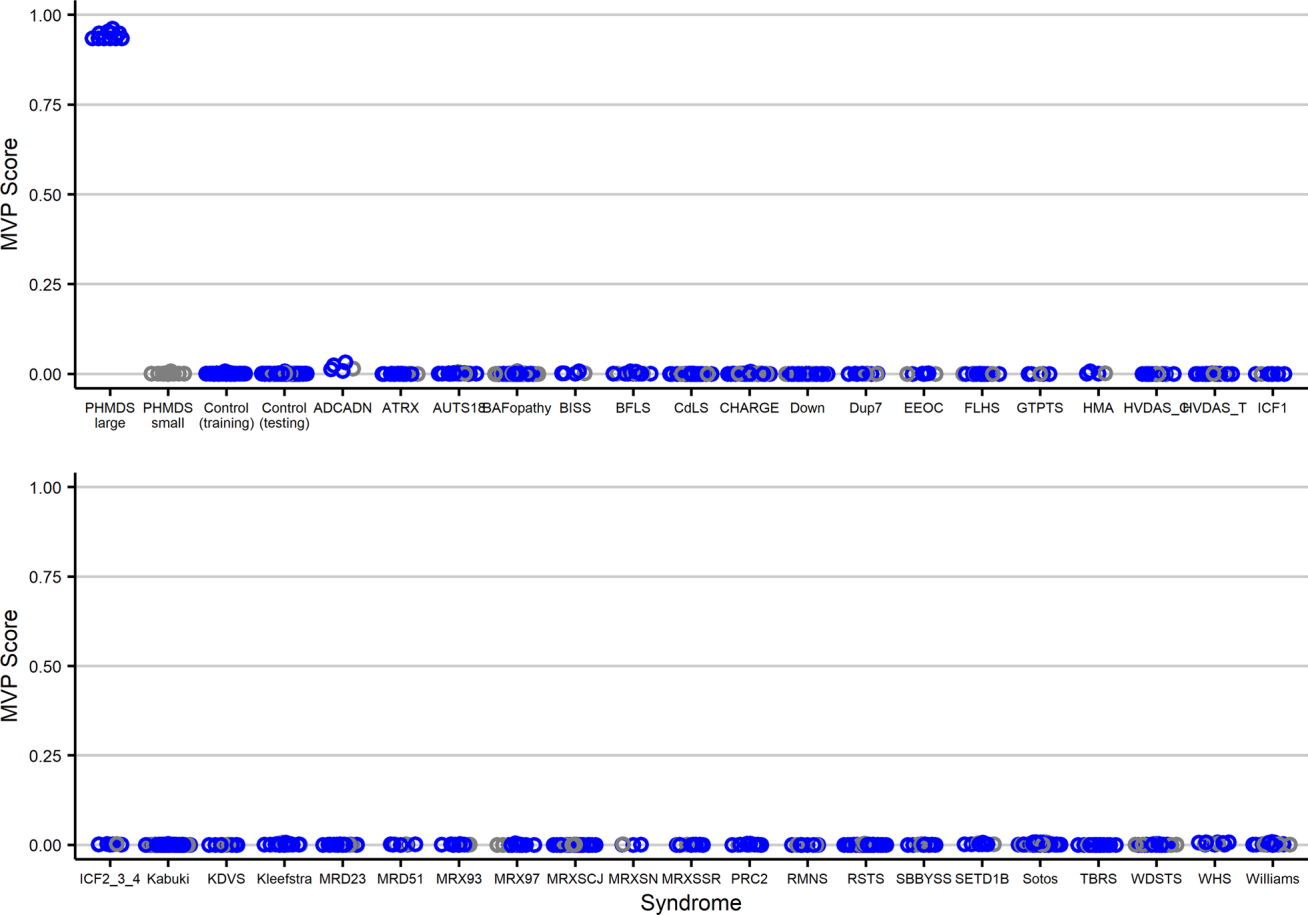
PHMDS methylation variant pathogenicity (MVP) score applied to PHMDS samples, controls, and over 1500 samples from patients with other neurodevelopmental syndromes. A SVM classifier was used to generate a score from zero to one for each subject as the probability of having a DNA methylation profile similar to what is observed in the PHMDS epi-signature. The Y-axis represents scores generated for each of the patient/control individuals on the X-axis. Every circle represents a single sample. The PHMDS large deletion samples, the matched controls used for probe selection and 75% of all other samples (controls and samples from patients with other neurodevelopmental syndromes) were used for training the classifier (blue). The PHMDS small deletion samples and remaining 25% of the controls and neurodevelopmental samples were used for testing (grey). Other neurodevelopmental conditions include subjects diagnosed with imprinting defects (Angelman, Prader-Willi, Beckwith-Wiedemann and Silver-Russell syndromes), non-syndromic autism spectrum disorders, BAFopathies, RASopathies, autosomal dominant cerebellar ataxia, deafness, and narcolepsy, ATRX, Borjeson-Forssman-Lehmann syndrome, Coffin-Lowry, Cornelia de Lange, CHARGE, Claes-Jensen, Down, Dup7, Floating-Harbor, Fragile X, Genitopatellar, Kabuki, Kleefstra, Rett, Sotos, Weaver, Cerebellar ataxia, deafness, and narcolepsy, immunodeficiency-centromeric instability-facial anomalies syndrome 1, Epileptic encephalopathy, Koolen–De Vries syndrome, SBBYSS syndrome, Rubinstein-Taybi syndrome 1, Rhaman syndrome, Helsmoortel-van der Aa syndrome, Autosomal dominant Mental retardation, X-linked Mental retardation, Tatton-Brown-Rahman syndrome Wiedemann-Steiner syndrome and Williams syndromes

The 11 PHMDS subjects with large deletions (PHMDS-Large Del cohort) and 44 matched controls, plus 75% of the remaining controls and 75% of the other syndrome samples from our EpiSign Knowledge Database (EKD) were used for model training. We limited the analysis to probes shared by both EPIC and 450 k platforms (*n* = 399,092). Probes were filtered to those with a minimum of 10% methylation difference from controls. The final probe list was used to train a multi-class support vector machine (SVM) with linear kernel on the training cohort. The methylation variant pathogenicity (MVP) score was set to generate a single score from 0 to 1, with 1 being a methylation pattern similar to the case samples and 0 being a methylation patter similar to the control samples. The class obtaining the greatest score determined the epi-signature classification. A series of tests were performed to challenge the reliability of the model. For classifier testing we used the 11 PHMDS small deletion samples plus the remaining 25% of controls and other syndrome samples from our EKD. First, the entire PHMDS-Large Del cohort was classified by the model. The correct classifications were assigned to all subjects predicted to have PHMDS with typical large deletion, with scores close to 1 and significantly different from controls. PHMDS Small Del/Mut individuals were assigned a score close to 0, similar to controls. To measure the specificity of the classifier, we tested DNA methylation profiles from over 1500 subjects with a confirmed diagnosis of a neurodevelopmental disorders, including trinucleotide repeat expansion abnormalities, imprinting defect disorders, BAFopathies, Mendelian disorders of the epigenetic machinery, Down syndrome, as well as 185 subjects with non-syndromic ASD (Fig. 3). All samples were classified as controls, further confirming the specificity of the PHMDS-Large Del classifier.

### CNV assessment from EPIC array

Using the normalized raw methylated and unmethylated intensities from EPIC array, we were able to identify Copy Number Variants (CNV) in this patient cohort. Of the 17 individuals with known deletions at 22q13, including 2 individuals with SHANK3 deletion only, we were able to identify all deletions, except for the mosaic case (Table 3). The deletion sizes range from 0.013 to 5.98 Mb. Strong correlation of the genomic coordinates and deletion sizes from EPIC versus chromosome microarray (CMA) testing was observed, proving that EPIC array can accurately be used to detect microdeletions at 22q13 in addition to providing information regarding the epi-signature. The mosaic case is undetermined as we were unable to detect the deletion or the epi-signature. The level of mosaicism in this sample is unknown and could be under the limit of detection of EPIC array.

**Table 3:** CNV results from CMA and EPIC array

Molecular cohort	ID	CMA Start (hg19)*	CMA End (hg19)*	CMA length (Mbp)	EPIC Start (hg19)	EPIC End (hg19)	EPIC length (Mbp)
PHMDS-Small Del/Mut	MS2449	NA	NA	NA	NA	NA	NA
PHMDS-Small Del/Mut	MS2452	NA	NA	NA	NA	NA	NA
PHMDS-Small Del/Mut	MS2453	NA	NA	NA	NA	NA	NA
PHMDS-Small Del/Mut	MS2455	NA	NA	NA	NA	NA	NA
PHMDS-Small Del/Mut	MS2457	NA	NA	NA	NA	NA	NA
PHMDS-Small Del/Mut	MS2676	51,137,326	51,150,064	0.01	51,135,138	51,147,735	0.013
PHMDS-Small Del/Mut	MS2675	51,137,243	51,150,025	0.01	51,135,138	51,147,735	0.013
PHMDS-Small Del/Mut	MS2450	51,121,362	51,183,855	0.06	51,123,475	51,225,561	0.1
PHMDS-Small Del/Mut	MS2456	50,566,503	51,244,566	0.68	50,580,743	51,225,561	0.64
PHMDS-Small Del/Mut	MS2454	50,429,645	51,244,566	0.81	50,248,907	51,225,561	0.98
PHMDS-Large Del	MS2463	49,228,863	51,178,150	1.95	49,238,268	51,225,561	1.99
PHMDS-Large Del	MS2444	48,896,156	51,219,009	2.32	48,872,890	51,225,561	2.35
PHMDS-Large Del	MS2443	48,654,949	51,197,716	2.54	48,651,166	51,214,353	2.56
PHMDS-Large Del	MS2441	48,224,354	51,244,566	3.02	48,231,823	51,225,561	2.99
PHMDS-Large Del	MS2445	47,731,071	51,193,680	3.46	47,557,457	51,225,561	3.67
PHMDS-Large Del	MS2447	46,885,541	51,244,566	4.36	46,895,349	51,225,561	4.33
PHMDS-Large Del	MS2442	46,505,605	51,244,566	4.74	46,507,241	51,214,353	4.71
PHMDS-Large Del	MS2440	46,464,060	51,244,566	4.78	46,458,783	51,221,675	4.76
PHMDS-Large Del	MS2458	46,080,136	51,244,566	5.16	46,084,862	51,225,561	5.14
PHMDS-Large Del	MS2465	45,576,757	51,178,264	5.6	45,558,433	51,225,561	5.67
PHMDS-Large Del	MS2462	45,277,036	51,178,258	5.9	45,250,061	51,225,561	5.98
PHMDS Small Del/Mut *	MS2460	42,578,595	50,296,515	7.72	NA	NA	NA

NA (not available): samples with intragenic variants in *SHANK3* gene and mosaic PHMDS case. *Mosaic case. *CNV* copy number variants, *CMA* chromosome microarray analysis

### Identification of the PHMDS DNA methylation epi-signature critical genomic region

**Fig. 4:**
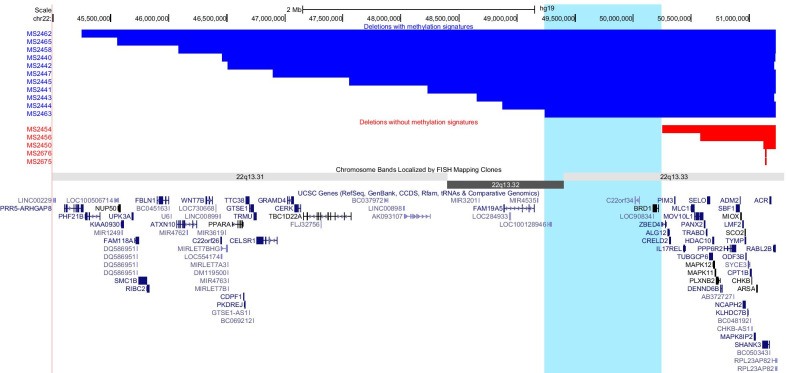
Phelan-McDermid syndrome deletions illustrating the genomic region of interest associated with methylation epi-signature. The horizontal blue bars represent deletions (2–6 Mb) associated with the presence of a distinct epi-signature. The horizontal red bars represent smaller deletions (0.01–1 Mb) that do not have a distinct methylation signature. Also shown in light blue is the common region of interest (22:49,238,268–50,248,907) in deletions associated with a methylation signature. All coordinates are taken from the EPIC Assay CNV analysis results. Cytogenetic bands and known genes are presented in this figure using the UCSC genome browser (Kent et al. 2002) 2009 (GRCh37/hg19) genome build

We next evaluated the genomic characteristics of PHMDS individuals that present with an epi-signature versus individuals without a signature. Figure 4 shows the genomic location of the 22q13 deletions in 16 individuals, of whom 11 presented with epi-signature (PHMDS-Large Del cohort), and 5 other who do not show epi-signature. This analysis lets us narrow down the critical genomic region (Chr22:49,238,268–50,248,907) shared between all subjects with epi-signature and not shared with subjects without the epi-signature. The only fully contained protein-coding gene in the PHMDS DNA methylation epi-signature critical region is *BRD1* (bromodomain-containing protein 1; OMIM# 604589). In addition, this critical region of overlap contains the transcript *C22orf34* and exon 1 of *ZBED4* (NM_014838.2), as well as the non-protein-coding RNAs *LOC100128946* and *LOC90834*.

### Metabolic profiling

When compared to 50 control lymphoblastoid cell lines (LCLs), the 9 cell lines from individuals with small 22q13 deletions (< 1 Mb) or pathogenic variants of *SHANK3* showed only one well that reached statistical significance according to adjusted *p* value: α-d-glucose from the tryptophan (Trp) plate (adjusted *p* value = 0.006). However, in 11 cell lines from individuals with large 22q13 deletions (> 1 Mb), 340 out of the total 776 wells (43.8%) reached an adjusted *p* value < 0.05. Of these 340 wells, all but two (containing sodium chloride at two different concentrations, adjusted* p* values = 0.027 and 0.033) showed reduced NADH production in the samples with large deletions (Additional file 3: Figure S3, Table 4, Additional file 5: Table S4, Aditional file 6: Table S5).

**Table 4:** Selected significant compounds identified by metabolic profiling of cell lines from the PHMDS (*n* = 11) and PHMDS Small Del/Mut (*n* = 9) cohort

Compound (PM plate)	Difference with controls	*p* value
PHMDS Small Del/Mut		
α-d-Glucose (PM-Trp)	Lower	0.001
PHMDS		
d-Fructose-6-Phosphate (PM-M1)	Lower	7.26E-05
d-Glucose-1-Phosphate (PM-M1)	Lower	5.38E-04
d-Glucosaminic acid (PM-M1)	Lower	2.45E−03
d-Galactose (PM-M1)	Lower	2.03E−03
β-Hydroxy-butyric acid (PM-M1)	Lower	3.53E−03
γ-Amino-butyric acid (PM-M1)	Lower	7.51E−03
α-d-Glucose (PM-M1)	Lower	0.014
Propionic acid (PM-M1)	Higher	0.014
d-Glucose-6-Phosphate (PM-M1)	Lower	0.017
d-Mannose (PM-M1)	Lower	0.017
*N*-acetyl-neuraminic acid (PM-M1)	Lower	0.019
α-Keto-butyric acid (PM-M1)	Lower	0.019
Pyruvic acid (PM-M1)	Lower	0.026
Ala-Leu (PM-M2)	Lower	0.001
Ala-Trp (PM-M2)	Lower	0.002
Arg-Phe (PM-M2)	Lower	0.002
His-Tyr (PM-M3)	Lower	0.007
His-Trp (PM-M3)	Lower	0.007
His-Val (PM-M3)	Lower	0.014
l-Tryptophan (PM-Trp)	Lower	9.37E−03
Trp-Gly (PM-Trp)	Lower	2.61E−03
Trp-Lys (PM-Trp)	Lower	6.34E−03
Triiodothyronine (PM-M6, well E3)	Lower	2.33E−05
Epinephrine (PM-M6, well C3)	Lower	9.92E−05
Progesterone (PM-M6, well G1)	Lower	1.51E−04
Resistin (PM-M7, well B5)	Lower	0.0002
hGH (Somatotropin) (PM-M7, well E3)	Lower	0.0002
FGF-1 (PM-M7, well F1)	Lower	0.0002
IL-1β (PM-M7, well G1)	Lower	0.0002

For the selected relevant compounds, we considered trends based on unadjusted *p* values to highlight the clustering of metabolic findings suggestive of specific pathways

Assessment of individual plates showed that no particular patterns emerged in the small-deletion cohort, except for a reduced capacity of utilizing amino acids as energy sources, including tryptophan from the Trp plate, lower levels of NADH in the presence of 3-isobutyl-1-methylxanthine (3 out of 6 wells) and higher levels in the presence of sodium chloride (3 out of 6 wells).

Alternatively, the large deletion cohort showed several patterns of disrupted metabolic pathways. The PM-M1 plate showed a pattern of reduced utilization as energy source of the main carbohydrates—α-d-Glucose (*p* = 0.01), confirmed also by the data from the Trp plate (*p* = 0.00007), fucose (*p* = 0.007), galactose (*p* = 0.002), mannose (*p* = 0.017), dextrin (*p* = 0.029)—and their phosphorylated or methylated forms, of intermediates of the Krebs cycle like pyruvic (*p* = 0.026) and a-keto-butyric acids (*p* = 0.019), ketone bodies like acetoacetic (*p* = 0.027) and β-hydroxy-butyric acid (*p* = 0.003) and molecules with important roles in the nervous tissue, such as *n*-acetyl-neuraminic acid (*p* = 0.018) and γ-amino-butyric acid, or GABA (*p* = 0.007). The large deletion cohort showed no significant differences in the utilization of alternative energy sources. PM-M2 to M4 and Trp plates in the large-deletion cohort demonstrated a pattern of generalized reduction of the utilization of amino acids and dipeptides as energy sources, with over-representation of large amino acids, such as Trp, Phe, Tyr, Val, Leu and Ile (*p* < 0.05). Exposure to many ionic species caused reduced NADH levels in cells carrying large 22q13 deletions, particularly the ones containing sodium, namely sodium chloride (*p* = 0.0001 to 0.045), sodium tungstate (*p* = 0.00007 to 0.007), sodium phosphate (*p* = 0.0006 to 0.003), sodium pyrophosphate (*p* = 0.00006 to 0.0003) and sodium nitrate (*p* = 0.0003 to 0.008), and the ones containing chloride, such as lithium chloride (*p* = 0.0008 to 0.01), ferric chloride (*p* = 0.007 to 0.049), ammonium chloride (*p* = 0.0003 to 0.007), zinc chloride (*p* = 0.018 to 0.046), copper chloride (*p* = 0.001 to 0.007) and cobalt chloride (*p* = 0.0007 to 0.017). Overall 171 out of 288 wells (59.4%) from the combined PM-M6 to M8 plates showed reduced energy production in cells carrying large 22q13 deletions (adjusted *p* values < 0.05). Among the metabolic effectors showing the most significantly abnormal values there were epinephrine (*p* = 0.0001 to 0.0008), steroids and related regulatory hormones, like progesterone (*p* = 0.0001 to 0.002), dexamethasone (*p* = 0.0003 to 0.01), 4,5-α-dihydrotestosterone (*p* = 0.001 to 0.005), LH (*p* = 0.0009 to 0.007) and LH-RH (*p* = 0.006 to 0.024), hormones involved in the thyroid function and regulation, such as triiodothyronine (*p* = 0.00002 to 0.005), TSH (*p* = 0.0009 to 0.01) and TRH (*p* = 0.016 to 0.035), hormones with anabolic or pro-digestive effects, like resistin (*p* = 0.0002 to 0.005), ghrelin (*p* = 0.0003 to 0.002) and gastrin (*p* = 0.0004 to 0.007), growth factors, like somatotropin (*p* = 0.0002 to 0.002) and fibroblast growth factor 1, FGF-1 (*p* = 0.0002 to 0.002), and cytokines, such as IL-1β (*p* = 0.0002 to 0.005), IL-2 (*p* = 0.007 to 0.03), IL-6 (*p* = 0.002 to 0.023) and IFN-γ (*p* = 0.012 to 0.031).

## Discussion

This study describes a novel genome-wide DNA methylation epi-signature associated with PHMDS. By assessing a patient cohort with variable breakpoints within the 22q13.3 locus and individuals with *SHANK3* mutations, we identified a critical region including the *BRD1* gene which is required for the PHMDS epi-signature. Individuals with DNA methylation epi-signature showed significantly different metabolomic profiles when compared to the epi-signature negative individuals with small deletions sparing BRD1 and with *SHANK3* mutation, indicating evidence of two clinical subtypes within PHMDS that are distinct at the molecular and phenotypic levels.

The genomic region associated with PHMDS varies in size and contains multiple protein-coding genes as well as miRNAs and other non-coding RNAs. Deletions, duplications and mutations involving the *SHANK3* gene within the chromosomal region 22q13.33 are considered to be responsible for many of the neurological findings that can be seen in PHMDS subjects [2, 7, 8, 29]. Severity of phenotype has been correlated with an increased deletion size in multiple studies [8, 13, 30]. However, there are reports of smaller deletions in subjects with some features of a more severe phenotype including expressive speech delay [31], as well as interstitial deletions not involving the *SHANK3* gene in subjects with clinical features of PHMDS [11]. These and many additional studies have suggested that other, more proximal genes, are responsible for the severe phenotypic features observed in individuals with larger deletions of 22q13 [4, 11, 13, 32]. Currently, it is unclear whether other genes within the 22q13 region contribute to the neurological and additional phenotypic features of the disorder. Our findings show that only individuals with large deletions (2 to 6 Mb) of 22q13 have a distinct epigenetic signature despite the disruption of *SHANK3* in all individuals (Fig. 4). This suggest that SHANK3 is not responsible for the methylation alterations observed in PHMDS.

The common region of overlap within the large deletion cohort contains the genes *BRD1*, *C22orf34* and the first exon of *ZBED4*, as well as the non-protein-coding RNAs *LOC100128946* and *LOC90834*. Of these genes the bromodomain containing protein 1 (*BRD1*) gene at chromosome region 22q13.33 is the most likely candidate for the observed genome-wide DNA methylation defects observed in these individuals. *BRD1* is a component of a histone acetyltransferase complex that can stimulate acetylation of histone H3 [33, 34] through interactions with chromatin remodeling proteins, e.g., PBRM_1_ and histone modifiers, e.g., MYST2 and SUV_420_H1 [33]. Disruption of histone acetylation mechanisms are consistent with our findings showing predominant DNA hypermethylation in PHMDS individuals whose deletions include the *BRD1* gene.

Currently there is a limited genotype–phenotype association of *BRD1* loss in individuals with PHMDS. In one study focused on identification of 22q13 genes most likely to contribute to PHMDS, *BRD1* was estimated to result in a high loss of function intolerance in these individuals [17]. This gene has been implicated in susceptibility to schizophrenia and bipolar disorders due to its effect on transcriptional regulation of numerous genes and brain development [33, 35–37]. Although psychiatric presentation of PHMDS is not well characterized, there are a few cases reported with schizophrenia and other psychiatric features [38, 39].

Evaluation of the differentially methylated regions identified in this cohort of individuals with large deletions at 22q13 indicates several genes that may play a role in the pathophysiology of this syndrome (Additional file 4: Table S3). For example, the *LAMA1* gene is associated with autosomal recessive Poretti-Boltshauser syndrome, characterized by cerebellar dysplasia, myopia, variable retinal dystrophy and eye movement abnormalities ataxia, delayed motor development, language impairment and intellectual disability. *IFT140* is associated with autosomal recessive Mainzer-Saldino syndrome, a disorder characterized by kidney disease, eye problems and skeletal abnormalities. *EBF4* and *ARPP21* are both involved in brain development and neurotransmission. Further studies are necessary to evaluate the effect of methylation change on transcriptional activity of the related proteins and their impact on the clinical phenotype in PHMDS.

Consistent with genome-wide disruption in DNA methylation, metabolic profiling of subjects with the large 22q13 deletions including *BRD1* gene showed a clearly recognizable metabolic profile, characterized, among the other features, by a reduced capacity of the cells to produce energy in the presence of high-efficiency energy sources, a decreased ability to adjust to metabolic environments influenced by different concentrations of ionic species and an abnormal response to hormones and cytokines involved in regulating growth, proliferation, energy storage and inflammation. PHMDS subjects with the small 22q13 deletions sparing *BRD1* gene or with *SHANK3* pathogenic variants are metabolically undistinguishable from controls. The two cohorts shared only a reduced utilization of amino acids as energy sources, although the values were more significant in the cases with large deletions. This metabolic feature has been reported in cases with isolated ASD [40, 41], and all 20 individuals tested by the Biolog arrays presented autistic traits; therefore, it is possible to infer that in individuals with PHMDS the presence of autism as well as the disruption of the metabolic pathways involved in amino acids utilization are not influenced significantly by the size of the 22q13 deletion. Taken together, our findings demonstrated that individuals with large deletions encompassing BRD1 gene present with robust epigenetic and metabolic alterations. The resulting metabolic alteration may be caused by either or both the epigenetic alteration, which can alter the expression of critical metabolic genes, and/or loss of a larger genetic material, which may contain genes for metabolic pathways.

Comparison of the clinical features presented by the individuals with PHMDS in the PHMDS Large Del versus the PHMDS Small Del/Mut cohorts showed no significant differences in the most common traits, involving neurobehavioral issues, such as ASD or autistic traits and other behavioral or mental disorders, and other neurological signs and symptoms, such as seizures, sleep disorders and hypotonia (Table 3). These findings are in line with the widely accepted mechanistic model suggesting a central role for *SHANK3* haploinsufficiency or disruption in the pathogenesis of most neurological features of PHMDS [4, 7, 10]. Other neurological traits such as abnormal regulation of body temperature and high tolerance to pain seem to be more frequent among individuals with large 22q13 deletions, but clinical data about these traits were available only for a limited number of individuals. Finally, non-neurological problems, involving gastrointestinal, renal and muscular/skeletal systems, resulted more represented in the cohort with large deletions, although also in these cases information was available for a limited number of cases. As observed in the metabolic findings, this greater involvement of different systems in individuals with large 22q13 deletions is likely reflecting the disruption of more genes and, consequently, more pathways, due to the abnormal methylation mediated by the loss of *BRD1*.

Taken together, we showed that PHMDS individuals with the large 22q13 deletions including the *BRD1*-critical region present with robust metabolic, genomic and epigenomic alterations that likely contribute to a more severe and variable phenotype in PHMDS. Identification of individuals with loss of function mutations in the *BRD1* gene specifically will help more precisely determine the contribution of *BRD1* to the more complex range of presentations in the genetically heterogeneous PHMDS subtypes.

Neurodevelopmental disorders, including autism, schizophrenia, Down syndrome, Rett and Fragile X syndromes, Phelan-McDermid, Sotos, Kleefstra, Coffin Lowry and ATRX syndromes, and the disorders of imprinting, Angelman and Prader-Willi syndromes, are accompanied by aberrant epigenetic regulation of processes critical for normal and brain development [42]. However, the direct role of this methylation alteration on the pathophysiology of many of these diseases still needs further investigation. Our group has previously identified epi-signatures that can be used to specifically diagnose subjects with a variety of neurodevelopmental conditions, including syndromes that may clinically overlap with PHMDS [22]. The use of DNA methylation signatures can help solve many clinically ambiguous cases presenting with a neurodevelopmental phenotype.

While whole-genome chromosome microarray and sequencing techniques remain the standard approach for assessment of individuals with suspected PHMDS, a genomic DNA methylation array may be used to augment this approach. Previous studies have highlighted the ability to detect copy number variants in DNA methylation arrays at comparable sensitivity to other established methods, e.g., chromosomal microarray [43–45]. We have shown the efficacy of applying computational methods to methylation data from the EPIC assay to determine copy number variants in individuals with PHMDS. There were no significant differences in the size or the coordinates identified by the EPIC assay versus the detection method used at time of diagnosis (Table 2). Implementation of whole genome methylation array in PHMDS diagnosis and potentially in other microdeletion syndromes will enable the identification of epi-signatures as well as determination of the deletion/duplication in a single test.

## Conclusion

The capability of the methylation array to detect both the methylation epi-signatures from a variety of clinically related neurodevelopmental disorders and copy number variants on the same array may have the potential to be applied as a more informative and cost-effective first-tier test to screen individuals with a broad range of developmental disorders. In addition, the identification of a PHMDS epi-signature in an individual may suggest the development of specific PHMDS phenotypes with a more severe and variable presentation, including risk for schizophrenia. Finally, investigation of the genes affected by the abnormal DNA methylation may lead to the identification of novel targets for more personalized treatment approaches.

## Material and methods

### Study cohort

This patient cohort included 22 individuals, 11 males and 11 females, with diagnosis of PHMDS referred for genetic testing at the Greenwood Genetic Center, USA, and Federico II University, Italy. The current diagnosis criteria for Phelan-McDermid syndrome include both clinical findings and detection of a heterozygous deletion of chromosome 22q13.3 with involvement of at least part of SHANK3, or a heterozygous pathogenic variant in SHANK3 on molecular genetic testing (https://www.ncbi.nlm.nih.gov/books/NBK1198/). The clinical criteria for the diagnosis include developmental and language delay, hypotonia, autistic traits and/or other behavioral problems, seizures, sleep disturbances and minor dysmorphic traits. The cases were reviewed by Drs. Curtis Rogers and Luigi Boccuto, who possess vast experience in PHMDS. After initial molecular/cytogenetic assessment, 15 individuals had 22q13 deletions, one of which was mosaic, with deletion sizes ranging from 0.06 to 5.87 Mb, 2 individuals with a *SHANK3* gene deletion (0.013 Mb) and 5 individuals with *SHANK3* loss-of-function pathogenic variants. Ages spanned from 3 to 19 years, with a median age of 10 years and a mean age of 10.3 years.

Genomic methylation analysis was performed on peripheral blood-extracted genomic DNA. Samples were subdivided into two cohorts: typical PHMDS cohort (referred to as PHMDS-Large Del), including 11 cases with large (> 1 Mb) deletions at 22q13; and small deletion/*SHANK3* mutation cohort (referred to as PHMDS Small Del/Mut), including 10 cases with 22q13 deletions smaller than 1 MB and SHANK3 intragenic mutations. A mosaic case with typical deletion was included in the PHMDS Small Del/Mut cohort.

The set of controls that were used for mapping the epi-signatures, feature selection and model training were chosen from our EpiSign Knowledge Database (EKD). Genomic DNA methylation profiles from individuals with other congenital syndromes that are commonly involved on the differential diagnosis of PHMDS were also used for assessment of the specificity of the epi-signature (EpiSign Knowledge Database [22]) and included a cohort of individuals with Prader-Willi syndrome and Angelman syndrome, Fragile X syndrome, Rett syndrome, FG syndrome 1, Sotos syndrome and ASD.

Any subject used herein to represent a condition had a confirmed clinical diagnosis of the aforementioned syndrome and was screened for mutations in the related genes. The mutation report from every individual was reviewed according to the American College of Medical Genetics and Genomics (ACMG) guidelines for interpretation of genomic sequence variants [46], and only individuals confirmed to carry a pathogenic or likely pathogenic mutation together with the clinical diagnosis were used to represent a syndrome.

### Cytogenetic analysis

Genetic rearrangements were measured from whole blood DNA specimens by chromosome microarray analysis (CMA) using a custom 4 × 44 K 60-mer oligo array designed to cover 22q12.3-terminus by Oxford Gene Technology (Oxford, UK) as described by Sarasua et al. [13]. CMA genomic coordinates of breakpoints were established according to the 2006 human genome build (NCBI 36/HG 18) and converted from hg18 to hg19 using the UCSC LiftOver tool [47].

### Sanger sequencing

Sequencing of *SHANK3* was completed using standard Sanger sequencing protocol including coding sequences ± 20 nucleotides intronic. All variants identified by direct sequencing were analyzed using available bioinformatic websites to assess their potentially deleterious effect on the proteins and classified according to ACMG guidelines [46].

### Methylation array and quality control

DNA methylation protocol, analysis and epi-signature construction were performed according to our previously published protocol [22, 23, 48, 49]. Peripheral whole blood DNA was extracted using standard techniques. Following bisulfite conversion, DNA methylation analysis of the samples was performed using the Illumina Infinium methylation EPIC bead chip arrays (San Diego, CA), according to the manufacturer’s protocol. The resulting methylated and unmethylated signal intensity data were imported into R 3.5.1 for analysis. Normalization was performed using the Illumina normalization method with background correction using the minfi package [50]. Probes with detection *p* value > 0.01, those located on chromosomes X and Y, those known to contain SNPs at the CpG interrogation or single nucleotide extension, and probes known to cross-react with chromosomal locations other than their target regions were removed, resulting in 753,265 probes remaining for the analysis. Arrays with more than 5% failure probe rate were excluded from the analysis. Sex of the subjects was predicted using the median signal intensities of the probes on the X and Y chromosomes, and those samples discordant between the labeled and predicted sex were not used for analysis. All of the samples were examined for genome-wide methylation density, and those deviating from a bimodal distribution were excluded. Factor analysis using a principal component analysis (PCA) was performed to examine the batch effect and identify the outliers.

### Selection of matched controls for methylation profiling

For mapping the epi-signature (probe and feature selection), matched controls were randomly selected from our EpiSign Knowledge Database (EKD). All of the PHMDS samples were assayed using the EPIC array. Therefore, all controls selected for epi-signature identification were analyzed using the same array type (EPIC). Matching was done by age, sex and batch using the MatchIt package. The control sample size was increased until both the matching quality and sample size were optimized and consistent across all analyses. This led to the determination of a control sample size four times larger than that of the cases in every analysis. Increasing the sample size beyond this value compromised the matching quality. After every matching trial, a PCA was performed to detect outliers and examine the data structures. Outlier samples and those with aberrant data structures were removed before a second matching trial was conducted. The iteration was repeated until no outlier sample was detected in the first two components of the PCA.

### DNA methylation profiling of Phelan-McDermid syndrome

The methylation level for each probe was measured as a beta value, calculated from the ratio of the methylated signals versus the total sum of unmethylated and methylated signals, ranging between zero (no methylation) and one (full methylation). This value was used for biological interpretation and visualization. For linear regression modeling, beta values were logit transformed to *M* values using the following equation: log2(beta/(1 − beta)). A linear regression model using the limma package [51] was used to identify the differentially methylated probes. The analysis was adjusted for blood cell-type compositions, estimated using the algorithm developed by Houseman et al. [52]. The estimated blood cell proportions were added to the model matrix of the linear models as confounding variables. The generated *p* values were moderated using the eBayes function in the limma package and were corrected for multiple testing using the Benjamini and Hochberg method. Probes with a corrected *p* value < 0.01 and a methylation difference greater than 10% were considered significant. The effect size cutoff of 10% was chosen to avoid reporting of probes with low effect size and those influenced by technical or random variations as conducted in our previous studies [23, 49]. The analysis was repeated 3 times: 22 PHMDS samples with 88 matched controls, 11 PHMDS-Large Del with 44 matched controls, 11-Small Del with 44 matched controls. Because only the cohort of individuals with PHMDS with large deletions (> 1 MB) showed significant methylation difference, this and the following steps were performed using only the cohort with large deletions (referred to as PHMDS Large Del).

### Clustering and dimension reduction

Following every analysis, the selected probes were examined using a hierarchical clustering and a multiple dimensional scaling to examine the structure of the identified epi-signature. Hierarchical clustering was performed using Ward’s method on Euclidean distance by the gplots package. Multiple dimensional scaling was performed by scaling of the pair-wise Euclidean distances between the samples.

### Leave-2-out cross-validation using PHMDS large deletion samples

For each round of validation, nine of the eleven PHMDS large deletion samples were used for probe selection along with matched controls and the remaining two PHMDS large deletion samples were saved for testing. Multidimensional scaling was used to cluster the samples. This was repeated 55 times for each combination of pairs of PHMDS-Large Del samples.

### Identification of the differentially methylated regions

To identify genomic regions harboring methylation changes (differentially methylated regions—DMRs), the DMRcate algorithm was used [28]. First, the *p* values were calculated for every probe using multivariable limma regression modeling. Next, these values were kernel smoothed to identify regions with a minimum of three probes no more than 1 kb apart and an average regional methylation difference > 10%. We selected regions with a Stouffer transformed false-discovery rate (FDR) < 0.01 across the identified DMRs. The analysis was performed on the same sets of cases and controls used for methylation profiling and was adjusted for blood cell-type compositions. Gene ontology analysis with the differentially methylated genes was performed using http://www.webgestalt.org/.

### Construction of a classification model for Phelan-McDermid syndrome

A classification model, referred to as methylation variant pathogenicity (MVP) score, was created to assess the specificity of the identified methylation signature using all of the identified probes. We trained a support vector machine (SVM) with linear kernel on the PHMDS-Large Del cases and controls. For classifier/model training we compared the 11 PHMDS-Large Del samples to the same 44 controls from probe selection, plus 75% of the remaining controls and 75% of the other syndrome samples from our EKD. For classifier testing we used the 11 PHMDS small deletion samples plus the remaining 25% of controls and other syndrome samples from our EKD. The EKD samples include both EPIC and 450 K samples to ensure the classifier works with both array types. Given the majority of the samples to be tested later were assayed using 450 k array, we limited the analysis to probes shared by both array types. Training was done using the e1071 R package. To determine the best hyperparameter used in linear SVM (cost—C), and to measure the accuracy of the model, tenfold cross-validation was performed during the training. In this process, the training set was randomly divided into tenfold. Ninefold was used for training the model and onefold for testing. After tenfold repeating of this iteration, the mean accuracy was calculated, and the hyperparameters with the most optimal performance were selected. For every subject, the model was set to generate a score ranging 0–1, representing the confidence in predicting whether the subject has a DNA methylation profile similar to PHMDS-Large Del. Conversion of SVM decision values to these scores was done according to the Platt’s scaling method [53]. The class obtaining the greatest score determined the predicted phenotype. A classification as PHMDS was made when a sample received the greatest score for that class (normally greater than 0.5). The final model was applied to both training a large cohort of individuals with other neurodevelopmental disorders as well as a group of healthy controls to determine the specificity of the signature.

### Validation of the classification model

We ensured that the model is not sensitive to the batch structure of the methylation experiment by applying it to all of the samples assayed on the same batch as the cases used for training. To confirm that the classifier is not sensitive to the blood cell-type compositions, we downloaded methylation data from isolated cell populations of healthy individuals from GEO (GSE35069) [54], supplied them to the classification model for prediction and examined the degree to which the scores were varied across different blood cell types. Next, the model was applied to the patient cohort to evaluate the predictive ability of the model on affected subjects. To determine the specificity of the model, we supplied a large number of DNA methylation arrays from healthy subjects to the model. To understand whether this model was sensitive to other medical conditions presenting with neurodevelopmental disorders and intellectual disabilities, we tested a large number of subjects with a confirmed clinical and molecular diagnosis of such syndromes by the model. These subjects are part of the EpiSign Knowledge Database housed at London Health Sciences and include > 5000 individuals with various neurodevelopmental disorders and non-affected controls. Information about this database can be found in our previous publications [22, 23].

### Copy number variation (CNV) analysis using methylation array data

To estimate the copy number alterations in the PHMDS samples from the infinium methylation array, the raw methylated and unmethylated intensities from every PHM *This may reflect the difficulty* DS sample and the same number of controls from the same batch were summed, and quantile normalized using the preprocessCore package (Bioconductor.org). The normalized matrix values in all samples were divided by the median values of every probe across the normal samples. The divided ratios were then log10 transformed, smoothed and segmented using the DNAcopy Bioconductor package (Bioconductor.org) to identify genomic regions in every sample showing a copy number change. A *p* value of < 0.01 obtained from 10,000 permutations was used to define a change point during segmentation. Neighboring segments with an average difference in ratio of < 0.05 were joined before a visual comparison of the ratio plots, and the identified breakpoints led to the determination of the CNV coordinates.

### Metabolic profiling

#### Lymphoblastoid cell lines (LCLs)

Peripheral blood samples were collected from 20 individuals with PHMDS by venipuncture (individuals MS2676 and MS2675 were excluded from this analysis). Lymphoblastoid cell lines (LCLs) were obtained by immortalization via Epstein-Barr virus of lymphocytes isolated from the blood samples. The lymphoblastoid cell lines were harvested in Sigma RPMI-1640 with 15% fetal bovine serum (FBS) from Atlanta Biological (Flowery Branch, GA, USA) and 2 mM l-Glutamine, 100 U/mL Penicillin and 100 µg/mL Streptomycin from Sigma-Aldrich (St. Louis, MO, USA).

#### Metabolic profiling via Biolog Phenotype Mammalian MicroArrays (PM-Ms).

Metabolic profiling was measured to assess impact of position effects on metabolism. The Phenotype Mammalian MicroArray (PM-M) developed by Biolog (Hayward, CA, USA) is designed to measure the cellular production of NADH (nicotinamide adenine dinucleotide, reduced form) in the presence of different compounds. Lymphoblastoid cell lines were used to measure metabolic dysregulation in Biolog Metabolic Arrays. These cell lines generated from the patient’s blood sample via Epstein-Barr virus transfection were counted utilizing a TC20™ Automated Cell Counter in order to measure the amount and the percentage of viable cells. The methodology employs 96-well microplates with diverse molecules, which act either as energy sources (plates PM-M1 to M4) or as metabolic effectors (plates PM-M5 to M8). Each well contains a single chemical, and production of NADH per well is monitored using a colorimetric redox dye chemistry. The energy sources include carbohydrates, nucleotides, carboxylic acids and ketone bodies in plate PM-M1, amino acids, both alone and as dipeptides (plates PM-M2 to M4). The metabolic effectors include ions (PM-M5), hormones, growth factors and cytokines (PM-M6 to M8). These metabolic effectors (PM-M5-M8) were tested in different concentrations for each compound (Additional file 6: Table S5). The custom tryptophan plate (Trp) generated by Biolog in collaboration with Greenwood Genetics Center (GGC) was also employed in consideration of previously published data showing decreased utilization of tryptophan as energy source by cells from individuals with ASD [41]. This plate is constituted by twelve 8-well columns containing glucose, empty well, tryptophan alone and 5 dipeptides in which tryptophan is combined, respectively, with glycine, lysine, leucine, arginine and alanine. Overall, 776 wells were analyzed for each cell line: 96 for each plate from PM-M1 to PM-M8 (768) and 8 from the PM-Trp plate. A list of chemicals used in the 776 wells is included in Additional file 5: Table S4. PM-M plates were incubated with 20,000 lymphoblastoid cells per well (40,000/well for the Trp plate) in a volume of 50 μl, using the modified Biolog IF-M1 medium. The medium for plates PM-M1 to M4 was prepared by adding the following to 100 mL of Biolog IF-M1: 1.1 mL of 100 × penicillin/streptomycin solution, 0.16 mL of 200 mM Glutamine (final concentration 0.3 mM) and 5.3 mL of fetal bovine serum (final concentration 5%). For the Trp plate 1.1 mL of fetal bovine serum was added instead of 5.3 mL, for a final concentration of 1%. For plates PM-5 to M8, 5.5 mL of 100 mM glucose (final concentration 5%) was added in place of the fetal bovine serum. The cells were incubated for 48 h at 37 °C in 5% CO_2_. After this first incubation, Biolog Redox Dye Mix MB was added (10 μL/well) and the plates were incubated under the same conditions for an additional 24 h, during which time the cells metabolize the sole carbon source in the well. As the cells metabolize the carbon source, tetrazolium dye in the media is reduced, producing a purple color according to the amount of NADH generated. During the 24 h of exposure to the dye, the plates were incubated in the Omnilog system, which measured the optical density of each well every 15 min, generating 96 data points. The information collected during the 24 h was analyzed by the kinetic software of the system to generate kinetic curves of the NADH generation for each well and calculate kinetic parameters, such as slope, endpoint and area under the curve. At the end of the 24-h incubation, the plates were analyzed utilizing a microplate reader with readings at 590 nm and 750 nm. The first value (A_590_) indicated the highest absorbance peak of the redox dye, and the second value (A_750_) gave a measure of the background noise. The relative absorbance (A_590–750_) was calculated per well.

For Phenotype Mammalian data, the absorbance endpoint readings and the 96 datapoints of kinetic optical density collected over the 24 h of incubation with the tetrazolium dye in the Omnilog system were used for data normalization and statistical analysis using R (opm R package) [55]. The readings were normalized using the triplicate absorbance readings from the corresponding empty plate (plates run with no cells, just media and dye). These values were then transformed to a logarithmic scale for the analysis and compared versus the average values generated by 50 lymphoblastoid cell lines from healthy controls. The control samples were obtained from peripheral blood samples of 50 typically developing subjects from North and South Carolina (USA), 24 males and 26 females (male-to-female ratio 0.92), whose age at blood sampling ranged from 1.2 to 10.3 years. Our goal was to identify the wells in which the levels of NADH generated by PHMDS cells were significantly different from the ones measured in controls. We utilized the R package to implement the nonparametric Mann–Whitney approach of a two-sided *t* test with the cutoff of *p* value ≤ 0.05 (https://www.rdocumentation.org/packages/stats/versions/3.6.2/topics/wilcox.test). The generated *p* values were corrected for multiple testing using the Benjamini and Hochberg method using the R package (https://www.rdocumentation.org/packages/stats/versions/3.6.2/topics/p.adjust) to obtain adjusted *p* values. The Mann–Whitney test was performed for the large- and small-deletion cohort against the controls separately*. *Using this approach, we were able to identify the metabolites differentially metabolized between cases with PHMDS and controls.

### Web resources

Phelan-McDermid syndrome foundation, https://www.PHMDSf.org/registry/

Bioconductor, https://bioconductor.org/

Online Mendelian Inheritance in Man, https://www.omim.org/

GEO DataSets, https://www.ncbi.nlm.nih.gov/gds

## Supplementary Information

**Additional file 1:** **Table S1.** Differentially methylated probes in PHMDS samples compared to controls. For each probe, listed are: methylation % difference, p value, adjusted p value, chromosome and chromosome position, and where available overlapping UCSC Gene name, UCSC gene type and UCSC CpG island.

**Additional file 2:** **Table S2.** PHMDS and control sample information. Details for each sample listed include: age, sex, and the predicted cell type composition.

**Additional file 3: Figure S1.** Leave-2-out cross-validation using PHMDS large deletion samples. For each round of validation, nine of the eleven PHMDS large deletion samples were used for probe selection along with matched controls and the remaining two PHMDS large deletion samples were saved for testing. Multidimensional scaling was used to cluster the samples. Each time the two testing samples clustered with the other PHMDS samples. This was repeated 55 times for each combination of pairs of PHMDS large deletion samples, the first 12 are shown here. **Figure S2.** Gene Ontology enrichment analysis was performed using WEB-based GEne SeT AnaLysis Toolkit. Minimum number of IDs in the category: 3. Among the 24 unique genes, 15 were annotated to the selected functional categories, which are used for the enrichment analysis. Based on the above parameters, 3 positive related categories and 5 negative related categories are identified as enriched categories and all are shown in this report. **Figure S3.** Graphical representation of the metabolic profiles in PHMDS and PHMDS Small Del/Mut cell lines versus controls. The figure shows kinetic curves generated by optical density readings collected every 15 minutes for 24 hours, for a total of 96 data-points for each well. Plates PM-M5 to M8 are represented, average data from cases, either the 11 PHMDS cell lines or the 9 PHMDS Small Del/Mut ones, are in green while average data from 50 control cell lines are in red, the area where case and control data overlap is shown in yellow. Therefore, red edges indicate lower NADH levels in case cell lines as compared to controls for the compound in the give well, and green edges indicate higher NADH levels in case cell lines than in controls. The metabolic profiles of the PHMDS Small Del/Mut cohort show almost exclusively yellow curves, suggesting a substantial overlap of case and control data. On the other hand, the numerous red edges and the few green ones (limited to PM-M5) in the PHMDS cohort indicate numerous differences as compared to controls in the production of NADH when exposed to different metabolic effectors. Overall, these graphics illustrate a normal metabolic profile of cell lines from individuals of the PHMDS Small Del/Mut cohort as opposed to a largely disrupted one in the cells from the PHMDS cohort.

**Additional file 4:** **Table S3.** PHMDS DMRs: Details for each DMR listed include: chromosome, start and end, width, number of CpGs, statistical significance (Stouffer and Fisher test), distance to nearest CpG island, distance to nearest gene and where available overlapping gene/s.

**Additional file 5:** **Table S4.** Metabolic profiling plates. Table includes listing of substrates in the individual wells of the metabolic profiling plates.

**Additional file 6:** **Table S5.** Statistical comparison of metabolite profiles. Results of the statistical comparisons for metabolite levels comparing control lymphoblastoid cell lines (LCLs) to the cell lines from individuals with small 22q13 deletions (< 1 Mb) or pathogenic variants of SHANK3 and to the cell lines from the individuals with large deletions. These include mean metabolite levels, p value, and adjusted p value.

## Data Availability

All relevant data are available within the article and the supplementary files.

## Abbreviations

ASD: Autism spectrum disorder
BRD1: Bromodomain-containing protein 1
CNV: Copy number variants
DMR: Differentially methylated regions
LCLs: Lymphoblastoid cell lines
MVP: Methylation variant pathogenicity
PCA: Principal component analysis
PHMDS: Phelan-McDermid syndrome
SHANK3: SH3 and multiple ankyrin repeat domains 3
Small Del/Mut: Small deletion/mutation
SVM: Support vector machine
Trp: Tryptophan

## References

[1] Bonaglia MC, Giorda R, Beri S, De Agostini C, Novara F, Fichera M (2011). Molecular mechanisms generating and stabilizing terminal 22q13 deletions in 44 subjects with Phelan/McDermid syndrome. PLoS Genet.

[2] Phelan K, McDermid HE (2012). The 22q13.3 deletion syndrome (Phelan-McDermid Syndrome). Mol Syndromol.

[3] Boccuto L, Lauri M, Sarasua SM, Skinner CD, Buccella D, Dwivedi A (2013). Prevalence of SHANK3 variants in patients with different subtypes of autism spectrum disorders. Eur J Hum Genet.

[4] Soorya L, Kolevzon A, Zweifach J, Lim T, Dobry Y, Schwartz L (2013). Prospective investigation of autism and genotype–phenotype correlations in 22q13 deletion syndrome and SHANK3 deficiency. Mol Autism.

[5] Leblond CS, Nava C, Polge A, Gauthier J, Huguet G, Lumbroso S (2014). Meta-analysis of SHANK mutations in autism spectrum disorders: a gradient of severity in cognitive impairments. PLoS Genet.

[6] Oberman LM, Boccuto L, Cascio L, Sarasua S, Kaufmann WE (2015). Autism spectrum disorder in Phelan-McDermid syndrome: initial characterization and genotype-phenotype correlations. Orphanet J Rare Dis.

[7] De Rubeis S, Siper PM, Durkin A, Weissman J, Muratet F, Halpern D (2018). Delineation of the genetic and clinical spectrum of Phelan-McDermid syndrome caused by. Mol Autism.

[8] Wilson HL, Wong AC, Shaw SR, Tse WY, Stapleton GA, Phelan MC (2003). Molecular characterisation of the 22q13 deletion syndrome supports the role of haploinsufficiency of SHANK3/PROSAP2 in the major neurological symptoms. J Med Genet.

[9] Bonaglia MC, Giorda R, Borgatti R, Felisari G, Gagliardi C, Selicorni A (2001). Disruption of the ProSAP2 gene in a t(12;22)(q24.1;q13.3) is associated with the 22q13.3 deletion syndrome. Am J Hum Genet.

[10] Jeffries AR, Curran S, Elmslie F, Sharma A, Wenger S, Hummel M (2005). Molecular and phenotypic characterization of ring chromosome 22. Am J Med Genet A.

[11] Wilson HL, Crolla JA, Walker D, Artifoni L, Dallapiccola B, Takano T (2008). Interstitial 22q13 deletions: genes other than SHANK3 have major effects on cognitive and language development. Eur J Hum Genet.

[12] Giza J, Urbanski MJ, Prestori F, Bandyopadhyay B, Yam A, Friedrich V (2010). Behavioral and cerebellar transmission deficits in mice lacking the autism-linked gene islet brain-2. J Neurosci.

[13] Sarasua SM, Dwivedi A, Boccuto L, Rollins JD, Chen CF, Rogers RC (2011). Association between deletion size and important phenotypes expands the genomic region of interest in Phelan-McDermid syndrome (22q13 deletion syndrome). J Med Genet.

[14] Sarasua SM, Dwivedi A, Boccuto L, Chen CF, Sharp JL, Rollins JD (2014). 22q13.2q13.32 genomic regions associated with severity of speech delay, developmental delay, and physical features in Phelan-McDermid syndrome. Genet Med.

[15] Sarasua SM, Boccuto L, Sharp JL, Dwivedi A, Chen CF, Rollins JD (2014). Clinical and genomic evaluation of 201 patients with Phelan-McDermid syndrome. Hum Genet.

[16] Tabet AC, Rolland T, Ducloy M, Lévy J, Buratti J, Mathieu A (2017). A framework to identify contributing genes in patients with Phelan-McDermid syndrome. NPJ Genom Med.

[17] Mitz AR, Philyaw TJ, Boccuto L, Shcheglovitov A, Sarasua SM, Kaufmann WE (2018). Identification of 22q13 genes most likely to contribute to Phelan McDermid syndrome. Eur J Hum Genet.

[18] Yousefi P, Huen K, Davé V, Barcellos L, Eskenazi B, Holland N (2015). Sex differences in DNA methylation assessed by 450 K BeadChip in newborns. BMC Genomics.

[19] Schenkel LC, Rodenhiser D, Siu V, McCready E, Ainsworth P, Sadikovic B (2017). Constitutional Epi/genetic conditions: genetic, epigenetic, and environmental factors. J Pediatr Genet.

[20] Martin-Herranz DE, Aref-Eshghi E, Bonder MJ, Stubbs TM, Choufani S, Weksberg R (2019). Screening for genes that accelerate the epigenetic aging clock in humans reveals a role for the H3K36 methyltransferase NSD1. Genome Biol.

[21] Jones MJ, Goodman SJ, Kobor MS (2015). DNA methylation and healthy human aging. Aging Cell.

[22] Aref-Eshghi E, Kerkhof J, Pedro VP, Barat-Houari M, Ruiz-Pallares N, Andrau JC (2020). Evaluation of DNA methylation episignatures for diagnosis and phenotype correlations in 42 Mendelian neurodevelopmental disorders. Am J Hum Genet.

[23] Aref-Eshghi E, Rodenhiser DI, Schenkel LC, Lin H, Skinner C, Ainsworth P (2018). Genomic DNA methylation signatures enable concurrent diagnosis and clinical genetic variant classification in neurodevelopmental syndromes. Am J Hum Genet.

[24] Schenkel LC, Aref-Eshghi E, Skinner C, Ainsworth P, Lin H, Paré G (2018). Peripheral blood epi-signature of Claes-Jensen syndrome enables sensitive and specific identification of patients and healthy carriers with pathogenic mutations in. Clin Epigenet.

[25] Aref-Eshghi E, Schenkel LC, Lin H, Skinner C, Ainsworth P, Pare G (2017). The defining DNA methylation signature of Kabuki syndrome enables functional assessment of genetic variants of unknown clinical significance. Epigenetics.

[26] Aref-Eshghi E, Schenkel LC, Lin H, Skinner C, Ainsworth P, Pare G (2017). Clinical validation of a genome-wide DNA methylation assay for molecular diagnosis of imprinting disorders. J Mol Diagn.

[27] Schenkel LC, Schwartz C, Skinner C, Rodenhiser DI, Ainsworth PJ, Pare G (2016). Clinical validation of fragile X syndrome screening by DNA methylation array. J Mol Diagn.

[28] Peters TJ, Buckley MJ, Statham AL, Pidsley R, Samaras K, Lord R (2015). De novo identification of differentially methylated regions in the human genome. Epigenet Chromatin.

[29] Macedoni-Lukšič M, Krgović D, Zagradišnik B, Kokalj-Vokač N (2013). Deletion of the last exon of SHANK3 gene produces the full Phelan-McDermid phenotype: a case report. Gene.

[30] Zwanenburg RJ, Ruiter SA, van den Heuvel ER, Flapper BC, Van Ravenswaaij-Arts CM (2016). Developmental phenotype in Phelan-McDermid (22q13.3 deletion) syndrome: a systematic and prospective study in 34 children. J Neurodev Disord.

[31] Luciani JJ, de Mas P, Depetris D, Mignon-Ravix C, Bottani A, Prieur M (2003). Telomeric 22q13 deletions resulting from rings, simple deletions, and translocations: cytogenetic, molecular, and clinical analyses of 32 new observations. J Med Genet.

[32] Ziats CA, Grosvenor LP, Sarasua SM, Thurm AE, Swedo SE, Mahfouz A (2019). Functional genomics analysis of Phelan-McDermid syndrome 22q13 region during human neurodevelopment. PLoS ONE.

[33] Fryland T, Christensen JH, Pallesen J, Mattheisen M, Palmfeldt J, Bak M (2016). Identification of the BRD1 interaction network and its impact on mental disorder risk. Genome Med.

[34] Christensen JH, Elfving B, Müller HK, Fryland T, Nyegaard M, Corydon TJ (2012). The Schizophrenia and bipolar disorder associated BRD1 gene is regulated upon chronic restraint stress. Eur Neuropsychopharmacol.

[35] Nyegaard M, Severinsen JE, Als TD, Hedemand A, Straarup S, Nordentoft M (2010). Support of association between BRD1 and both schizophrenia and bipolar affective disorder. Am J Med Genet B Neuropsychiatr Genet.

[36] Severinsen JE, Bjarkam CR, Kiaer-Larsen S, Olsen IM, Nielsen MM, Blechingberg J (2006). Evidence implicating BRD1 with brain development and susceptibility to both schizophrenia and bipolar affective disorder. Mol Psychiatry.

[37] Mishima Y, Miyagi S, Saraya A, Negishi M, Endoh M, Endo TA (2011). The Hbo1-Brd1/Brpf2 complex is responsible for global acetylation of H3K14 and required for fetal liver erythropoiesis. Blood.

[38] Messias E, Kaley SN, McKelvey KD (2013). Adult-onset psychosis and clinical genetics: a case of Phelan-McDermid syndrome. J Neuropsychiatry Clin Neurosci.

[39] Kohlenberg TM, Trelles MP, McLarney B, Betancur C, Thurm A, Kolevzon A (2020). Psychiatric illness and regression in individuals with Phelan-McDermid syndrome. J Neurodev Disord.

[40] Boccuto L, Chen CF, Pittman AR, Skinner CD, McCartney HJ, Jones K (2013). Decreased tryptophan metabolism in patients with autism spectrum disorders. Mol Autism.

[41] Cascio L, Chen CF, Pauly R, Srikanth S, Jones K, Skinner CD (2020). Abnormalities in the genes that encode Large Amino Acid Transporters increase the risk of Autism Spectrum Disorder. Mol Genet Genomic Med.

[42] Millan MJ (2013). An epigenetic framework for neurodevelopmental disorders: from pathogenesis to potential therapy. Neuropharmacology.

[43] Mah CK, Mesirov JP, Chavez L. An accessible GenePattern notebook for the copy number variation analysis of Illumina Infinium DNA methylation arrays. F1000Res. 2018;7:ISCB Comm J-1897. 10.12688/f1000research.16338.1. 10.12688/f1000research.16338.1 PMC6498745 31105932

[44] Feber A, Guilhamon P, Lechner M, Fenton T, Wilson GA, Thirlwell C (2014). Using high-density DNA methylation arrays to profile copy number alterations. Genome Biol.

[45] Cho S, Kim HS, Zeiger MA, Umbricht CB, Cope LM (2019). Measuring DNA copy number variation using high-density methylation microarrays. J Comput Biol.

[46] Richards S, Aziz N, Bale S, Bick D, Das S, Gastier-Foster J (2015). Standards and guidelines for the interpretation of sequence variants: a joint consensus recommendation of the American College of Medical Genetics and Genomics and the Association for Molecular Pathology. Genet Med.

[47] Hinrichs AS, Karolchik D, Baertsch R, Barber GP, Bejerano G, Clawson H (2006). The UCSC genome browser database: update 2006. Nucleic Acids Res.

[48] Aref-Eshghi E, Bend EG, Colaiacovo S, Caudle M, Chakrabarti R, Napier M (2019). Diagnostic utility of genome-wide DNA methylation testing in genetically unsolved individuals with suspected hereditary conditions. Am J Hum Genet.

[49] Aref-Eshghi E, Bend EG, Hood RL, Schenkel LC, Carere DA, Chakrabarti R (2018). BAFopathies' DNA methylation epi-signatures demonstrate diagnostic utility and functional continuum of Coffin-Siris and Nicolaides-Baraitser syndromes. Nat Commun.

[50] Aryee MJ, Jaffe AE, Corrada-Bravo H, Ladd-Acosta C, Feinberg AP, Hansen KD (2014). Minfi: a flexible and comprehensive Bioconductor package for the analysis of Infinium DNA methylation microarrays. Bioinformatics.

[51] Ritchie ME, Phipson B, Wu D, Hu Y, Law CW, Shi W (2015). limma powers differential expression analyses for RNA-sequencing and microarray studies. Nucleic Acids Res.

[52] Houseman EA, Accomando WP, Koestler DC, Christensen BC, Marsit CJ, Nelson HH (2012). DNA methylation arrays as surrogate measures of cell mixture distribution. BMC Bioinform.

[53] Smola A, Bartlett P, Schuurmans D, Schölkopf B (2000). Advances in large margin classifiers.

[54] Reinius LE, Acevedo N, Joerink M, Pershagen G, Dahlén SE, Greco D (2012). Differential DNA methylation in purified human blood cells: implications for cell lineage and studies on disease susceptibility. PLoS ONE.

[55] Vaas LA, Sikorski J, Hofner B, Fiebig A, Buddruhs N, Klenk HP (2013). opm: an R package for analysing OmniLog(R) phenotype microarray data. Bioinformatics.

