# Supplementary material for: DNA methylation epi-signature is associated with two molecularly and phenotypically distinct clinical subtypes of Phelan-McDermid syndrome
Source: Clin Epigenetics. 2021 Jan 6;13:2. doi: 10.1186/s13148-020-00990-7 (PMC7789817; doi:10.1186/s13148-020-00990-7)
Supplement: Supplementary file 3 — Additional file 3: Figure S1. Leave-2-out cross-validation using PHMDS large deletion samples. For each round of validation, nine of the eleven PHMDS large deletion samples were used for probe selection along with matched controls and the remaining two PHMDS large deletion samples were saved for testing. Multidimensional scaling was used to cluster the samples. Each time the two testing samples clustered with the other PHMDS samples. This was repeated 55 times for each combination of pairs of PHMDS large deletion samples, the first 12 are shown here. Figure S2. Gene Ontology enrichment analysis was performed using WEB-based GEne SeT AnaLysis Toolkit. Minimum number of IDs in the category: 3. Among the 24 unique genes, 15 were annotated to the selected functional categories, which are used for the enrichment analysis. Based on the above parameters, 3 positive related categories and 5 negative related categories are identified as enriched categories and all are shown in this report. Figure S3. Graphical representation of the metabolic profiles in PHMDS and PHMDS Small Del/Mut cell lines versus controls. The figure shows kinetic curves generated by optical density readings collected every 15 minutes for 24 hours, for a total of 96 data-points for each well. Plates PM-M5 to M8 are represented, average data from cases, either the 11 PHMDS cell lines or the 9 PHMDS Small Del/Mut ones, are in green while average data from 50 control cell lines are in red, the area where case and control data overlap is shown in yellow. Therefore, red edges indicate lower NADH levels in case cell lines as compared to controls for the compound in the give well, and green edges indicate higher NADH levels in case cell lines than in controls. The metabolic profiles of the PHMDS Small Del/Mut cohort show almost exclusively yellow curves, suggesting a substantial overlap of case and control data. On the other hand, the numerous red edges and the few green ones (limited to PM-M5) in the PHMDS coh [file 13148_2020_990_MOESM3_ESM.pptx]

## Slide 1
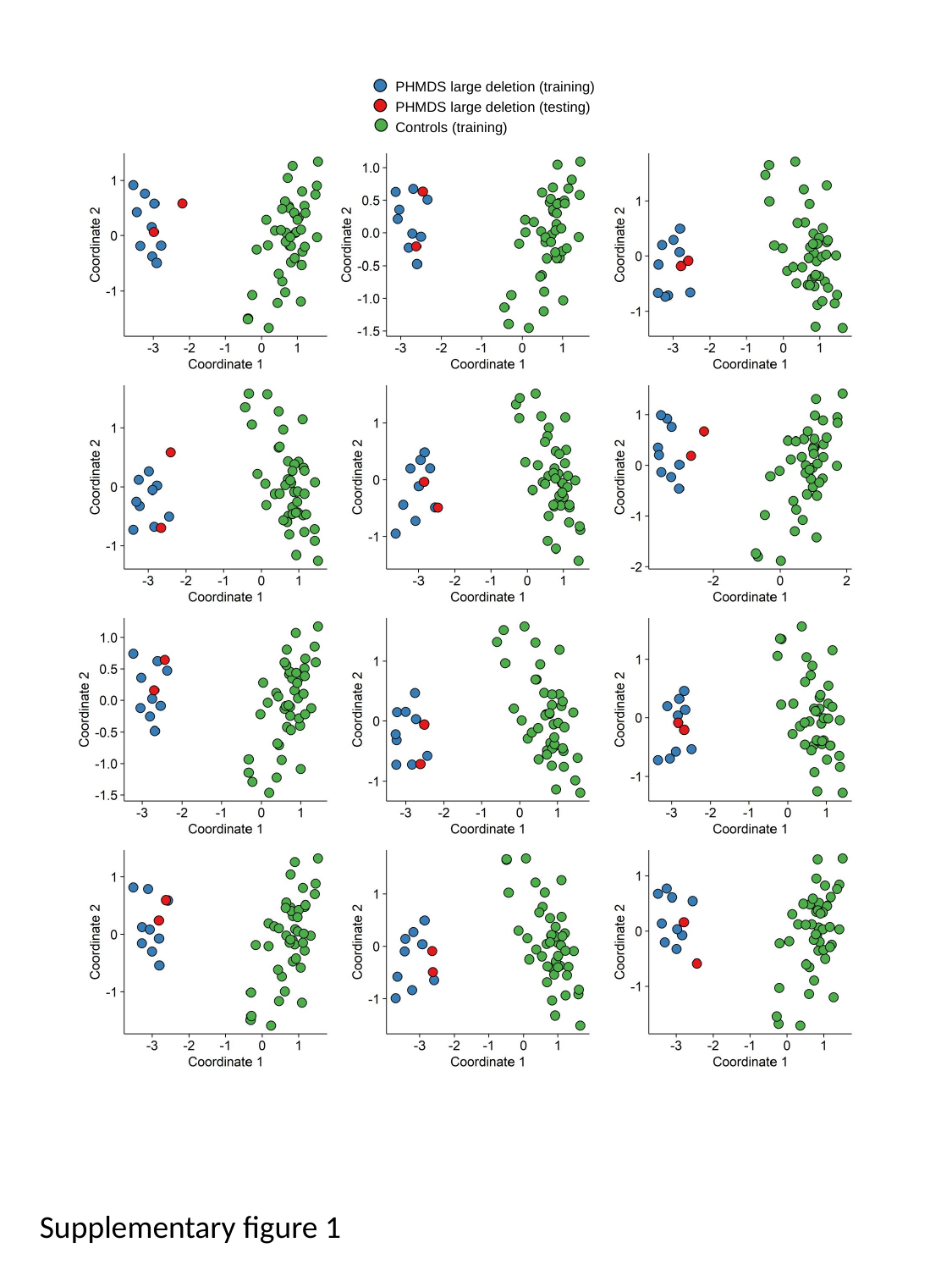

PHMDS large deletion (training)
PHMDS large deletion (testing)
Controls (training)
Supplementary figure 1

## Slide 2
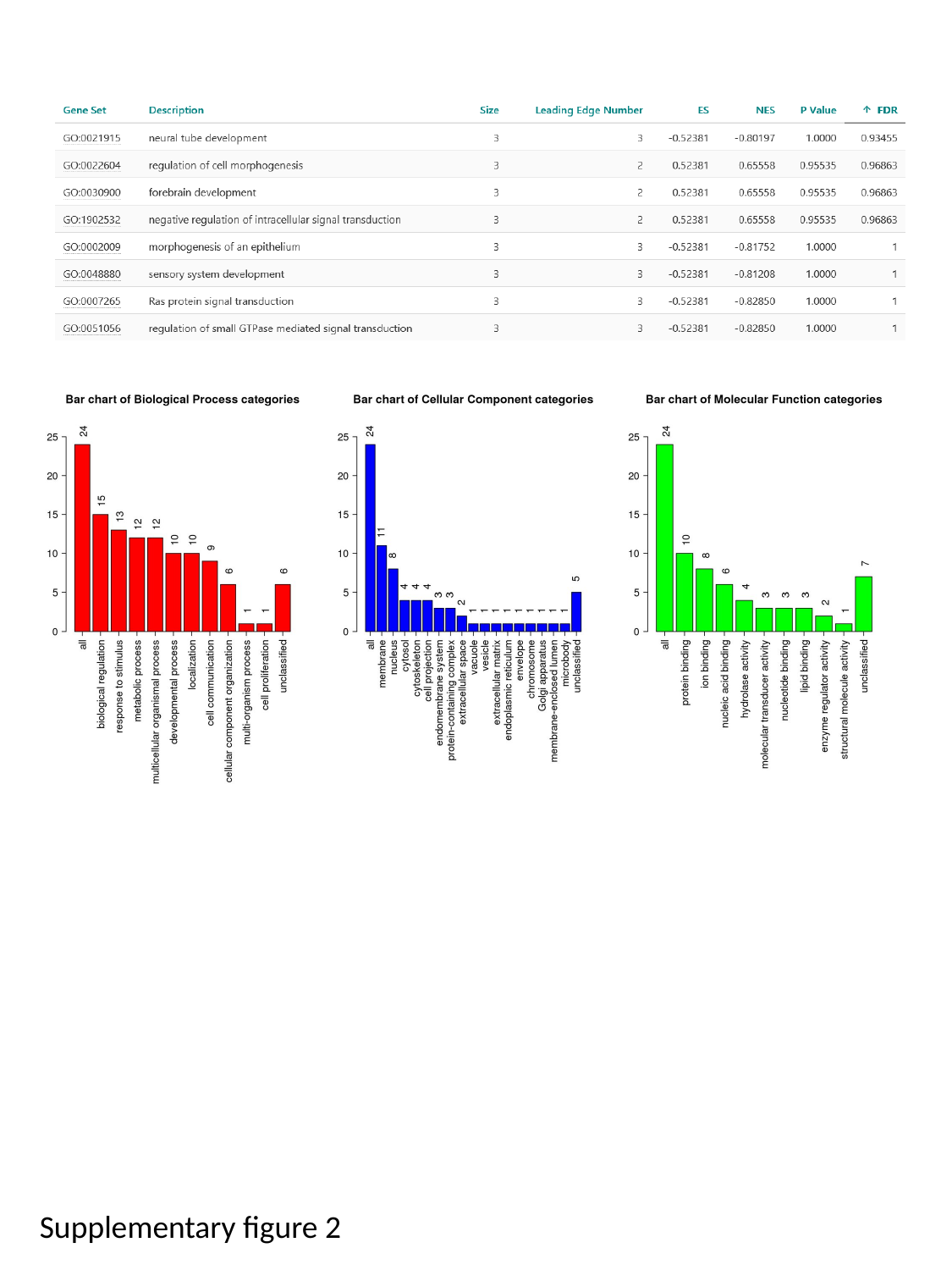

Supplementary figure 2

## Slide 3
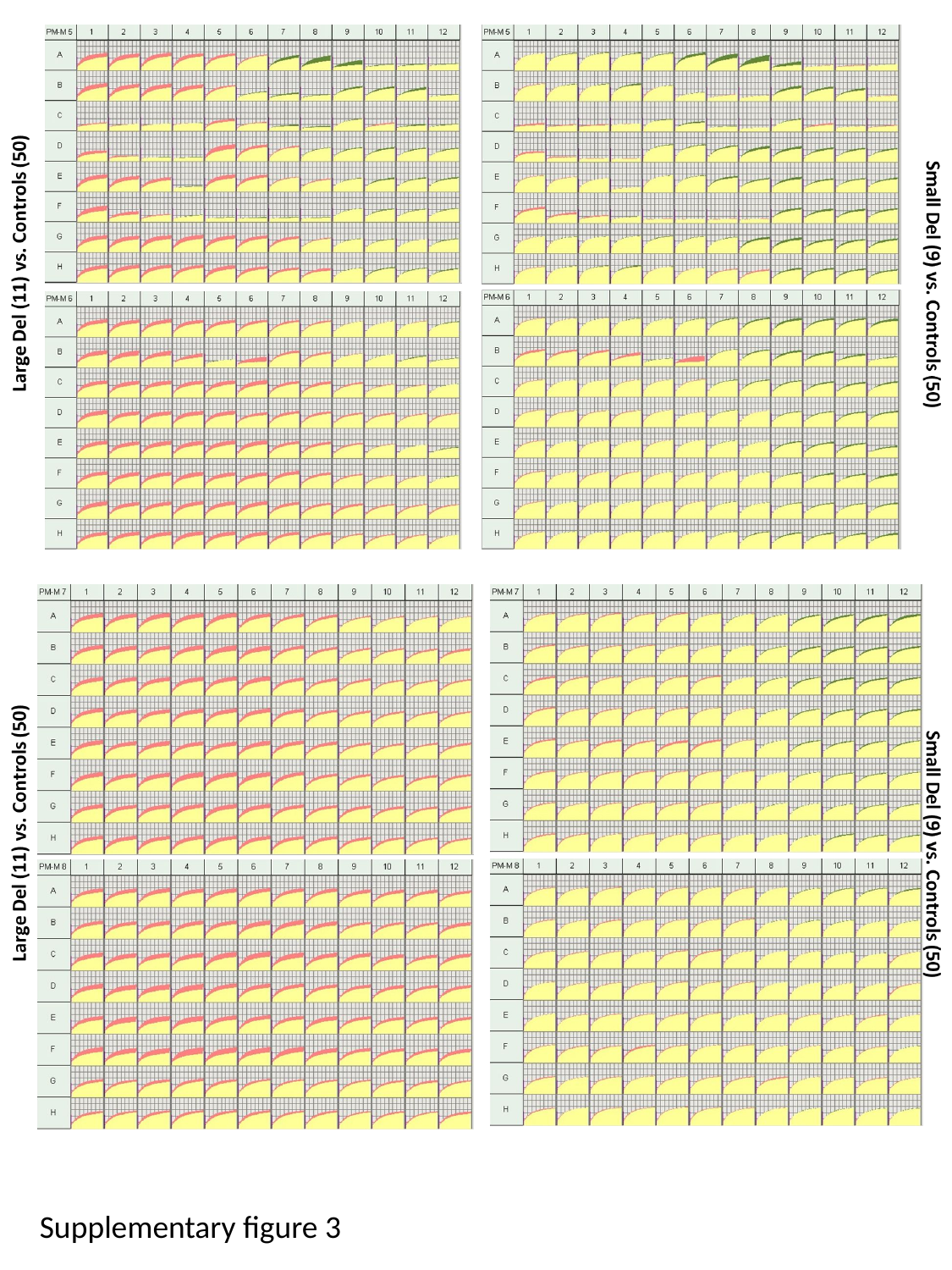

Supplementary figure 3
